# Ethiopian patients’ perceptions of anti-diabetic medications: implications for diabetes education

**DOI:** 10.1186/s40545-017-0101-2

**Published:** 2017-04-08

**Authors:** Bruck Messele Habte, Tedla Kebede, Teferi Gedif Fenta, Heather Boon

**Affiliations:** 1 School of Pharmacy, College of Health Sciences (CHS), Addis Ababa University (AAU), Addis Ababa, Ethiopia; 2 School of Medicine, CHS, AAU, Addis Ababa, Ethiopia; 3 Leslie Dan Faculty of Pharmacy, University of Toronto, Toronto, Canada

**Keywords:** Patients’ perceptions, Anti-diabetic medications, Qualitative interview, Necessity-concerns model, Ethiopia

## Abstract

**Background:**

The purpose of this study is to explore medication-related perceptions of adult patients with type 2 diabetes attending treatment in public hospitals of urban centers in central Ethiopia.

**Methods:**

Qualitative in-depth interviews were held with 39 participants selected to represent a range of treatment experiences and socio-demographic characteristics who were attending their treatment in 3 public hospitals. Interviews continued until key themes were saturated. The interview and analysis was guided by Horne’s necessity-concerns model.

**Results:**

The findings revealed medication-related perceptions some of which were similar to those of Western patients and others that seem to be informed by local socio-cultural contexts. Participants’ perceptions focused on the necessity of and concerns about their anti-diabetic medications, giving more emphasis to the latter. Concerns were expressed about both perceived and experienced adverse effects, inconveniences in handling the medications and access. It was evident that some of these concerns were exaggerated but could nevertheless negatively affect adherence to prescribed medications including resistance to initiate insulin with potential impact on health outcomes.

**Conclusions:**

Understanding patients’ perceptions of their medications is critical for developing a diabetes education program that considers local contexts and beliefs to enhance adherence. Education programs should consider patients’ concerns about medication adverse effects and reasons for use so as to improve their adherence and health outcomes.

## Background

Diabetes mellitus, especially type 2 (hereafter referred to as diabetes) is increasingly becoming an important public health problem in sub-Saharan Africa including Ethiopia with a prevalence estimate of 5.1%, and higher rates reported from the urban centers [1–4]. Ethiopian studies indicate that the majority of patients have poor health status including blood glucose levels well above recommended levels with a high proportion also having experienced micro- and macro-vascular diabetes-related complications [5–9]. One of the studies also reported uncontrolled diabetes leading to hospital admissions in a high proportion of surveyed patients [6]. A similar study reported that direct hospital costs for patients with diabetes was significantly higher compared to controls and that substantial a part of the cost was related to treatment for diabetes-related complications [10].

Among the mainstay of diabetes therapy are the anti-diabetic agents which greatly contribute to the control of blood glucose levels and associated micro- and macrovascular complications. The success of these agents, and other components of the treatment regimen, depends among other things on patients’ adherence to their recommended regimen so as to prevent acute complications and reduce long term complications [11]. However, adherence to medication regimens has been found to be a major challenge for patients with diabetes. Lower adherence to recommended medication regimens has been reported to have a negative impact on health outcomes and health services utilization such as emergency department visits, hospitalization and doctors’ visits [12].

The review paper by Capoccia et al. (2016) found that lower medication adherence was related to concerns about medication side effects and doubts about their necessity. This study also highlighted the influence of cultural variations in medication-related beliefs among different ethnic groups on adherence. These findings and others suggest the need for patient-centered approaches that consider patient preferences and address their specific medication-related concerns [12]. A number of approaches to improve adherence to anti-diabetic medications are reported including educational and pharmacy-driven interventions [11, 13, 14]. The most effective approaches appear to be those that provide proactive care, and where visits are coordinated among a team of healthcare providers, in either community or institutional settings. Furthermore, interventions should be based on the principles of patient-centered care that require patients’ active involvement in their self-management [11, 15]. The different interventions are based on studies of medication adherence to diabetes and influencing factors that have mostly been conducted in Western nations or minority groups living in Western countries. There is a dearth of literature from developing countries like Ethiopia which are facing an increasing burden from diabetes.

Among the widely reported conceptual models to assess patients’ medication related perceptions is the necessity-concerns framework which posits that patients’ treatment perceptions, especially those with respect to beliefs about necessity and concerns, will play an influential role when patients come face to face with decisions to take medications [16, 17]. Patients’ perceptions are likely different from that of their healthcare providers, and are thought to be based on their perceptions of the evidence about the benefits and risks [18].

The ‘necessity’ part of the Necessity-Concerns framework deals with the perceptions of personal need that patients may have for the medicines which may also be influenced by their beliefs about the efficacy of the medicines [16, 17]. The ‘concerns’ part deals with both concrete and abstract concerns related to medication-taking. The concrete experiences have to do with unpleasant symptoms such as side-effects and disruptions of daily life, while abstract concerns have to do with worries that regular use could lead to dependence or long term effects [16, 17]. In cases when the perceived necessity is strong and the perceived concerns are weak, patients tend to follow recommendations to adhere while strong perceived concerns and weaker necessity may lead to them not to adhere to treatments. Treatment perceptions may also lead to unintentional non-adherence if, for instance, patients who consider their medicine to be unimportant for their condition forget to take it. Apart from necessity and concerns perceptions, culture has also been reported to influence adherence to medications. It is also noteworthy that patients may not adhere due to reasons related to unintentional non-adherence(i.e., due to lack of resources) [17].

There are a limited number of articles focusing on adherence to anti-diabetic treatment in developing countries like Ethiopia. The few studies from Ethiopia report unacceptably low levels of adherence to recommended medications. The common factors cited for the low levels of adherence include: being on non-insulin drug regimen [19]; consulting traditional healers [19]; lack of financial resources [20]; perceived side effects [20]; experience of depressive symptoms [21]; complexity of medication regimens [21] and concerns about medications’ safety [22]. All of these were reported in quantitative studies and provide limited insight into patients’ perspectives of their medications which may be used to design patient-centered interventions to encourage adherence.

The aim of this study was to conduct a theory-guided, exploratory study using qualitative methods to elicit medication-related beliefs of type 2 diabetes patients who are following treatment in Addis Ababa (the largest urban center of Ethiopia) and Butajira (a town in Central Ethiopia that serves as center for the demographic surveillance site of Addis Ababa University) [23]. The Necessity-Concerns conceptual framework that has been widely used to study chronic patients was selected as the conceptual framework [17].

## Methods

This study is part of a larger study that utlized qualitative interview methods to gain in-depth understanding of issues surrounding patients’ medication-related perpceptions in urban Ethiopia. In-depth interviews with individual participants were conducted from December 2013 to March 2014 in locations that were mutually agreed by individual participants and the first author (BMH, a licensed pharmacist and PhD student trained in qualitative research methods) such as a quiet place in the hospital compound, participants’ homes, church compound, quiet spot in cafes, an academic office and a hospital office. Ethical approval (036/13/PSP) was obtained from the Institutional Review Board of the College of Health Sciences, Addis Ababa University.

### Study setting

Participants were selected from three public hospitals in central Ethiopia. Two of these, Tikur Anbessa Specialized Hospital (hereafter referred to as Tikur Anbessa) and Yekatit 12 Medical College Hospital (hereafter referred to as Yekatit 12) are located in Addis Ababa and serve a high number of patients with type 2 diabetes. The third hospital, Butajira General Hospital (hereafter referred to as Butajira) located in Butajira town located 135 kms away from Addis Ababa is the only public hospital in this town and was included to explore the perspectives of patients in a peri-urban area of central Ethiopia. Table 1 gives a brief description of the two settings.

**Table 1:** Description of study settings

	Addis Ababa	Butajira
Significance	Largest urban center in Ethiopia	Home to the demographic surveillance site of AAU
Population [48]	3.2 million	63 thousand
Ethnic groups [49]	Amhara (47%), Gamo (1.7%), Guragie (16.3%), Oromo (19.5%), Silte (2.9%), Tigrie (6.2%), others (6.4%)	Guragie (82%), others (18%)
Religions [49]	Orthodox Christianity (74.7%), Islam (16.2%), Protestant Christianity (7.8%), others (1.3%)	Islam (51.3%), Orthodox Christianity (39.6%) and Protestant Christianity (8.7%), others (0.4%)
Literacy [49]	85.3%	37.9%

Tikur Anbessa is currently the highest referral hospital in Ethiopia. During the study period, patients were seen in the Endocrinology Unit that was run by 3 endocrinologists and 2 endocrinology fellows working as consultants on a rotating basis, up to 6 Internal Medicine residents who were assigned to take primary roles in managing patients during their month-long attachments, 6 nurses and 1 recently recruited pharmacist.

Yekatit 12 is a general hospital that has recently started training doctors. The services provided for patients with diabetes were mostly in the general outpatient department (with no dedicated or separate diabetes care clinic) that was run by 4 general practitioners with complex cases referred to the medical referral clinic, run by internists on a rotating basis. In both of the above hospitals, patients would be ‘randomly’ assigned to a physician each time they come for their appointment.

Butajira, also a general hospital, serves patients with diabetes at a medical clinic separate from other outpatients. The clinic was run by a general practitioner and a nurse where patients meet the same physician and nurse each time they come for their monthly appointments for at least a 6-month period.

### Study participants recruitment

Patients with diabetes who were attending treatment in the selected hospitals during the study period who were 18 years and older, had been prescribed anti-diabetic medications for minimum of one year and had no known or overt psychiatric problems were eligible to participate. Being a healthcare professional was the only exclusion criterion. Participants were purposively selected to include a wide variety of patients in terms of socio-demographic characteristics (age, sex, educational level, marital status, employment status, religious affiliations and place of residence), income level, medication regimen and years since diagnosis. Participant recruitment was facilitated by the clinic nurses who identified the patients and provided initial information about the study which was followed up by the first author who provided further information and recruited eligible patients.

### Interview methods

Interviews were conducted in the Amharic language which is the official and widely used language in the study settings. The first author conducted the interviews (audio recorded with partcipants’consent) that ranged from 30 to 120 min. Sets of questions recommended by Kleinman et al. (1978) [24] and those from the necessity-concerns framework [16] were used to frame the interview guide (Appendix). Study participants were asked to discuss their treatment perceptions including their views on the necessity and concerns about their medication regimen. The interview guide was translated to Amharic and back to English to check the consistency before using the Amharic version.

### Data analysis

The interviews were transcribed verbatim into MS Word by an experienced assistant. The quality of the transcripts was checked by the first author by listening to randomly selected recordings from each study site while reading transcripts. Transcripts were read repeatedly to ensure good understanding before coding them to identify components of the necessity-concerns framework. Coding included looking for new themes emerging from the data that were unique. Interviews continued until all key themes were saturated (the point at which no new information was emerging) [25]. Analysis and interpretation was carried out by the first author in collaboration with the last author. Initial coding and categorization was done in Amharic followed by further analysis and interpretation which was done after translating key components relevant to the emerging themes into English. NVivo 10 was used to manage the data.

## Results

### Description of the study participants

Forty-five patients who met the eligibility criteria were identified during the study period of which 6 did not participate either due to personal reasons or because of problems with telephone communication. Of the 39 patients with diabetes who participated in the study, 24 were residents of Addis Ababa following treatment at Tikur Anbessa and Yekatit 12 and the remaining of Butajira town or its environs. Table 2 summarizes demographic characteristics of the study participants. As notable differences were not observed in the perceptions of participants from the different sites, the data are presented as a single set.

**Table 2:** Study participants’ characteristics (*n* = 39)

*Sex*	
Female	19
Male	20
*Age (years)*	
30-39	2
40-49	8
50-59	14
60-69	10
>70	5
*Educational status*	
Low education (illiterate or basic literacy)	16
Elementary complete	8
Secondary school complete	8
Post-secondary school education	7
*Occupation*	
Clerical work	7
Rents house	4
Small business	5
Farming	5
Pensioner	9
Unemployed	5
Others	4
*Payment for health services incl. medicines*	
Out of pocket	13
Government	23
Employer	3
*Diabetes duration (years)*	
1–5	10
6–10	14
11–15	7
16–20	4
21–25	4
*Treatment regimen*	
Oral:	
Glibenclamide + metformin	22
Glimepiride + metformin	1
Glibenclamide	1
Insulin	14
Insulin plus metformin	1

### Treatment perceptions

The findings revealed that most study participants didn’t know the names of their anti-diabetes medicines and instead labeled them using the Amharic terms for pill *(kinin)* and injection *(merfe).* Among the pills glibenclamide (a sulfonylurea), was labelled as the small or the thin one, while metformin (a biguanide), was the bigger or fatter one. A few, with better education, used brand names such as Daonil (a popular trade name for glibenclamide even if most actually took the generic versions), or Metformin. Participants’ treatment perceptions focused primarily on the necessity and concerns although it was apparent that increased emphasis was given to the concerns aspects of the medications and less to their necessity.

With regards to necessity perceptions towards anti-diabetic medications, they were mostly related to the health benefits and their efficacy. Generally, perceptions of necessity were stronger, with respect to insulin than for oral hypoglycemic agents:

*Now, if you stop your medicines [the pills] it could bring problems, may make you lose your eye (sight), paralyzed. (Male, 61 years, elementary school complete)*

*And so when it wouldn't go down (with the tablets), they changed it to the injection …. And it's done well, I now started to feel good inside. There is no weakness that used to be there, none of that … (Female, 60 years, high school complete)*

Participants’ concerns with regards to their anti-diabetic medications can be broadly categorized into: a) perceived concerns about the efficacy of the oral anti-diabetic agents; b) abstract concerns/worries of adverse effects; c) the actual experience of adverse effects; d) challenges with use and storage and; e) challenges with medicines availability or affordability.

### Concerns about the efficacy of oral anti-diabetic agents

The efficacy of the generic oral anti-diabetic medications available through the hospital pharmacy either for free or at low prices were questioned mostly in relation to their perceived lesser efficacy compared to the western brand medicines.

*The Indian ones (tablets) are useless and if the government tried to import the German ones, now that could be helpful for the patients; otherwise these are useless. But what other choice do we have? … (Male, 54 years, high school complete)*

### Worries about potential adverse effects of the anti-diabetic medicines

Worries were expressed about possible adverse effects of anti-diabetic medicines with hypoglycemia and gastritis cited as common concerns and as well about ‘too many’ medicines to a lesser extent. There were cases however where certain diabetes complications such as impotence and eye damage were attributed to be adverse effects of the medications on the one hand and the hypoglycemia due to the medications was ascribed to diabetes on the other.

*They say that it (diabetes) makes you fall and that you should always have candy or sugar on you. But how can I take sugar when I have been prohibited. My sugar has gone up and I have been told not to eat sweets and so how can I carry with me sugar? (Male, 61 years, elementary school complete)*

*I hear from professionals with whom I work that the medicine has many side-effects; the Daonil creates problems on the eye, metformin damages the stomach. … Yes it (the medicine) has many side-effects. (Male, 46 years, Diploma holder)*

Insulin was associated with a higher degree of severity because patients believed it was prescribed when the diabetes was not responding to other forms of treatment. This was perceived as evidence that one has perhaps neared the end of life and thus some patients became very emotional when discussing new insulin prescriptions:

*The very idea of injection disturbs one emotionally. It is also disturbing when discussed by the community. (Male, 46 years, Diploma holder)*

Different adverse effects have been associated with insulin such as falls, paralysis and weight gain. The expression of some had an emotional tinge to it revealing a strong feeling against insulin. On the other hand, a participant who earlier on had a serious concern which has since been replaced with strong expressions about the necessity of insulin was an interesting finding.

*Injections need great care; if the (sugar) levels go down there is the possibility of falling down and that’s what I think of. I don't think I will be able to get up again if I fall. I am hypertensive and such complications may make me paralyzed. (Female, 55 years, Diploma holder)*

*They said that I deserved injections after this (sugar levels wouldn’t go down with the tablets)… I was very startled (when they told me to start on injections). I didn't think I would be able to stand and walk. I was so fearful…. (But) it is only after the injections that I regained my identity and I became happy. (Female, 65 years, low education status)*

The influence of others, be it positive or negative, on insulin initiation was also reported. The positive aspects which participants observed or heard seem to have allayed some of their concerns while the negative aspects such as observing the scars and suffering apparently have negative influences towards initiating insulin.

*I hate it [insulin]. Even when I got diabetes I hoped not to become an insulin user. I don't know why. I sadden when I see her (my sister's) body. When I see her I don't feel as if I am ill. I just see her suffering. The places in her body which have been injected have scarred so much and created a pattern on its own…. and her body is like a sieve. (Male, 45 years, high school complete)*

### Experience of adverse effects

Study participants described their experiences of adverse effects they perceived as being associated with their oral anti-diabetic agents and insulin. The most common adverse effects associated with oral anti-diabetic agents were gastritis. In addition, some taking glibenclamide reported hypoglycemic feelings such as feeling dizzy and shivering.

Hypoglycemia was a major concern for those on insulin where shivering, dizziness, loss of consciousness and falls were reported. It was evident that the hypoglycemic incidents were puzzling, and led to emotional reactions for some of the study participants:

*My problem now is my fainting, it disturbs me. I say what if it (the fainting) happens while travelling on the road. Who would pick me up? Then my mother would no more have a child and my daughter wouldn't have a mother. That possible scenario disturbs me a lot. (Female, 46 years, high school complete)*

The hypoglycemic incidents above have led to life-threatening incidents and hospital emergency admissions for some study participants while it has led to extended treatment and absences from work for others.

Fear of pain associated with injections, which may directly impact adherence, was also identified by study participants as a concerning side effect.

*The injection indeed is difficult. I used to shiver and in the process hurt my legs. I had started injections one time. And so it was very painful and I also told myself of its painfulness. I imagined not about the efficacy of the medicine but of the bruise marks that it will leave on my body. (Female, 37 years, high school complete)*

### Challenges with use and storage

Perceptions about the need for stricter adherence required of insulin were cited as reasons to resist recommendations to start this medication:

*I didn't want the injection because they told me that you couldn't discontinue it from time to time (as is possible for the pills in case one has to go for holy water). (Male, 68 years, low education status)*

*I think the injection is a bit complicated as compared to ours especially with regards to time. Now we can take our medicines (pills) but can stay for 2 or even 3 h without eating but they would fall. If they say 8 o'clock, they have to take it exactly at 8 o'clock. (Male, 63 years, high school complete)*

The inconvenience of handling insulin especially in relation to its cold storage requirement was mentioned to restrict mobility:

*I find the injection to be very difficult because I am used to the tablet… It is more convenient (to transport and store even in rural areas). The injection needs a fridge and when you go out it is difficult (to use it) because if it is not placed in the fridge it will be damaged. And so if I go to my relatives who live far away, I could take my medicines in my pocket. (Male, 54 years, high school complete)*

### Challenges with the availability and affordability of the medicines

Concerns were expressed by some in relation to the availability and affordability of the prescribed medicines. For some, the concern is related to having to pay for the medicines as it was not affordable for them even to buy from the hospital pharmacy where generic medicines were available at low prices. For others it was the unavailability in the hospital pharmacy which might lead to paying even higher prices in the private outlets, or worse, having to buy low quality products from illegal sources.

*There are some people who inject expired medicines after buying it from private sources… Now if the medicine is not available at the hospital you have to buy it from outside. One time a friend of mine was offered to buy such medicine by some people who just rolled it up in a plastic bag and he encountered a problem. Now many people face such problems. (Male, 57 years, elementary school complete)*

*Yes sometimes medicines are unavailable (at the hospital). Now last time they were unavailable and we had to buy at the pharmacy (private). When one is available the other is not. There is a problem with medicines. (Male, 54 years, high school complete)*

## Discussion

This qualitative study of patient perceptions of anti-diabetic medications is a first from Ethiopia. Many participants perceived the medications’ necessity, with stronger belief in the efficacy of insulin compared to the oral agents. Given the increased emphasis on concerns about medications compared to their necessity, it is quite likely that these perceptions impact adherence to prescribed regimens and recommendations to initiate insulin. This seems to be in line with the necessity-concerns framework proposed by Horne et al. (2003) where such perceptions about necessity and concerns can lead to intentional non-adherence [16].

A range of concerns about the potential adverse effects of the oral agents were raised. These include concerns which were similar to those raised by Western patients that were mainly about adverse effects (e.g. hypoglycemia and gastritis) and to a lesser extent the number of medicines being taken [26, 27]. Medications-related concerns which are specific to this study include perceived adverse effects which are actually expected as complications of diabetes itself (e.g., impotence and eye problems) or relating hypoglycemia to the illness itself as a complication and not to the medication as adverse effects as also reported by another local quantitative study [28]. This signifies about the need to be open in assessing patient perceptions so that appropriate education about the diabetes and the drugs used to treat it may be offered. Such measures could minimize safety concerns which is one of the major barriers reported by local studies that have been reported to affect adherence to medications [20–22].

This study revealed hypoglycemia to be a major concern among some study participants on oral agents. Hypoglycemic incidents which included falls that were encountered personally or among their social circles could expectedly make patients to skip doses or make them reluctant to accept dose increments or additional medication. As most study participants were taking glibenclamide, the reported hypoglycemia, is not unexpected given its high hypoglycemic potential [29, 30]. The findings from a study done in Nigeria similarly reported hypoglycemia as one of the most common side effects and common reasons for non-adherence for those patients on glibenclamide [31]. Glibenclamide has further been associated with cardiac risks where one study reported how the initial treatment with glibenclamide was associated with a more than two-fold increase in the risk of coronary artery disease which was however reduced with newer sulphonylureas such as glimepiride and gliclazide [32]. The findings of the present study and supporting literature further highlight about the need by the government to consider alternative generic oral anti-diabetic agents available with comparable costs but less hypoglycemic potential such as glimepiride, another member of the sulfonylurea family [29].

There were others who expressed concern about perceived inefficacy of the generic oral anti-diabetic agents that were made available to patients for free or at low prices. Such type of concern about generic medicines is reported in another study conducted among patients attending treatment for diabetes in selected public and private health facilities in Addis Ababa. This study revealed that only 30% were comfortable taking any brand even if their physician recommended it and quality was one of their main concerns [33]. The concern towards generic medicines do not seem to be limited to the patients but also extends to the providers. For example a study reported that over one-third of community pharmacy clients and pharmacy personnel apparently had distrust on the approval system for generic medicines in Ethiopia [34]. This issue needs the attention of policy makers and healthcare providers to provide relevant education about generic medicines as such concerns may affect adherence [35].

On the other hand findings of a local study that evaluated the quality of different brands of amoxicillin capsules marketed in Ethiopia showed that most generic brands were not interchangeable with the innovator brand. A recommendation was made by that study for the reinforcement of the capacity of the drug regulatory body including strengthening of post marketing studies [36]. Another post marketing study done to assess quality of antibiotic, antimalarial and anti-tuberculosis medicines from selected sites in Asia, Africa (including Ethiopia) and South America reported that 848 (5.6%) of the total samples failed the quality test and furthermore 81 counterfeit medicines were reported, 13.6% of which were from Africa [37]. This highlights for the need to work to strengthen the regulatory system and ensure the quality of medicines at all levels of the supply chain so that efforts to increase access to affordable, generic medicines and improve the health outcomes of patients achieve intended outcomes.

With regards to insulin initiation, most of the concerns are similar to those reported elsewhere including insulin signifying an increased degree of illness severity and adverse effects mainly hypoglycemia [38]. Among the concerns that may be unique to this study is the perceived need for stricter adherence required with insulin compared to the oral agents which is perceived to be complicated by local religious healing practices such as use of holy water [39]. The use of holy water has been reported to lead to discontinuation of treatment according to studies done on other chronic illnesses [40, 41]. This issue needs to be assessed in a nonjudgmental approach. Patients need to be educated about the benefits of following recommended regimens and not discontinuing their treatment even if they intend to use holy water. Such attempts actually may need the support of higher level policy interventions as has been tried for HIV/AIDS treatment. In this regard collaborative work with religious leaders has endorsed the combined use of anti-HIV medications and holy water as compatible, encouraging the use of both types of treatment and contributed to increase in adherence to recommended regimens [40].

The issue of perception that insulin needs to be refrigerated at all times appears to be another potential problem in relation to its initiation as many may not possess refrigerators. If they are living in the rural areas or are to travel outside urban centers, accessing cold storage may be even more difficult [42]. Such conditions could thus be considered as credible reasons to resist recommendations to initiate insulin regimen by the patients. Healthcare providers should take time to assess their patients’ perceptions and contexts to discuss as needed about the importance of cold storage conditions for insulin, its utility for short and long term conditions and possible options to store especially for the spare insulin vials such as with relatives, friends or others who have a fridge. They should also stress the acceptability of storing insulin that is being used at room temperatures and that it should not impede acceptance of an insulin regimen [42–45].

It was also apparent from this study that some of those who expressed concerns about insulin were willing to accept an insulin regimen if it were recommended by their healthcare providers. This should be given due consideration in light of the reluctance to prescribe it by some providers citing strong patient resistance [38, 46].

Group diabetes education programs should be strengthened. Patients who have started on insulin and demonstrated improved health outcomes may have passed through the same anxiety as those patients who have concerns and so may play beneficial roles in such programs. The utility of other patients who are more experienced and ‘further along the treatment trajectory’ has been cited in reassuring patients who may be anxious about starting new treatments such as insulin [47]. It has to be stressed however that while group education may play a critical role in the care program of patients, they should not be a substitute for one to one education of patients by appropriately trained healthcare providers. Such individual-based educations would allow assessment of patients’ knowledge, beliefs and requisite skills with respect to their medications and facilitate a more tailored and patient-centered approach in educating and discussion to alleviate their concerns and improve their medication-taking behavior.

This study explored the perceptions of limited groups of patients with diabetes – those following their treatment at a public hospital in urban settings and consented to participate –and may not be representative of a larger and more diverse population. The findings may not be applicable to those who may have avoided biomedical treatment and also those attending treatment in the private health institutions who may be of higher socio-economic standing. This study however recruited patients of diverse socio-demographic backgrounds and having varied duration of illness which has allowed a rich and diverse information on their perceptions about anti-diabetic medicines which gave us a good insight. This study also gave the context for the majority of patients with diabetes who attend treatment in these urban centers which can be translated to other hospitals and patients with similar settings.

## Conclusions

Findings of this study are suggestive of the utility of the necessity-concerns framework in organizing the medication-related perceptions in our setting. While some of the participants’ anti-diabetic medications-related perceptions appeared to be similar to those expressed by Western patients, there were perceptions that were different including the exaggerated concerns towards oral agents and insulin which could potentially lead to intentional non-adherence and affect health outcomes. Diabetes education, be it individual or in groups, needs to consider local contexts such as patients’ religious backgrounds and their socio-economic contexts in addressing about the use of holy water, medication adverse effects and insulin storage among other things.

## Appendix

Interview guide

1. What treatment has been recommended to you by your healthcare provider(s)?
2. What are the most important results you hope to receive from this treatment?
3. What is your view regarding the necessity of the treatment regimen(s) recommended by your healthcare provider?
4. What are your concerns in relation to the anti-diabetic medications that are recommended by your healthcare provider?
5. If there is any other thing you would like to add in relation to our discussion on medications that have been recommended for your diabetes?
